# Protective Effects of Naringenin and Apigenin in Ameliorating Skin Damage via Mediating the Nrf2 and NF-κB Pathways in Mice

**DOI:** 10.3390/foods12112120

**Published:** 2023-05-24

**Authors:** Jie Li, Bingyong Mao, Xin Tang, Qiuxiang Zhang, Jianxin Zhao, Hao Zhang, Shumao Cui

**Affiliations:** 1 State Key Laboratory of Food Science and Resources, Jiangnan University, Wuxi 214122, China; 7210112021@stu.jiangnan.edu.cn (J.L.); maobingyong@jiangnan.edu.cn (B.M.); xintang@jiangnan.edu.cn (X.T.); zhangqx@jiangnan.edu.cn (Q.Z.); zhaojianxin@jiangnan.edu.cn (J.Z.); cuishumao@jiangnan.edu.cn (S.C.); 2 School of Food Science and Technology, Jiangnan University, Wuxi 214122, China; 3 National Engineering Research Center for Functional Food, Jiangnan University, Wuxi 214122, China

**Keywords:** naringenin, apigenin, antioxidative system, inflammation, signaling pathway

## Abstract

Naringenin and apigenin are common flavonoids derived from edible plants with the potential to alleviate inflammation and improve skin antioxidation. This study aimed to evaluate the effects of naringenin and apigenin on oleic acid-induced skin damage in mice and compare their underlying mechanisms of action. Triglycerides and non-esterified fatty acids were significantly decreased by naringenin and apigenin, while apigenin intervention resulted in a better recovery of skin lesions. Naringenin and apigenin improved the antioxidative abilities of the skin by increasing catalase and total antioxidant capacity levels and decreasing malondialdehyde and lipid peroxide levels. The release of skin proinflammatory cytokines, such as interleukin (IL)-6, IL-1β, and tumor necrosis factor α, was inhibited after naringenin and apigenin pretreatments, but naringenin only promoted the excretion of IL-10. Additionally, naringenin and apigenin regulated antioxidant defense and inflammatory response by activating nuclear factor erythroid-2 related factor 2-dependent mechanisms and suppressing the expression of nuclear factor-kappa B. In summary, naringenin and apigenin are prospective ingredients that contribute to the amelioration of skin damage by activating anti-inflammatory and antioxidative responses.

## 1. Introduction

*Citurs sinensis* L.Osbeck and *Oenanthe javanica* (Blume) DC are accepted almost around the world as the food sources of humans to provide nutritional and functional ingredients [1,2]. They are reported to contain an abundance of antioxidant nutraceuticals or phytochemicals that potentially regulate human metabolism with constant ingestion in a favorable manner [2,3]. Their flavonoid components have been extracted and reported to have a variety of pharmacological activities that prevent chronic and degenerative diseases and even improve skin conditions [3,4]. Naringenin derived from *C. sinensis* L.Osbeck has been reported to result in significant improvement in skin inflammation, skin damage, and skin oxidative reaction in a UVB induced-skin model [5,6,7]. Recent studies have also pointed out that naringenin has potential effects on the suppression of skin cancer in mouse skin tumor models [8]. Additionally, apigenin is the most common flavonoid found in the plant ingredients of *O. javanica* (Blume) DC that has been used in a wide variety of medicines, foods, and other fields [9,10]. In vitro studies have shown that apigenin has anti-inflammatory and skin-protective activities via regulating the phosphorylation of mitogen-activated protein kinase (MAPK) signaling molecules [11]. Apigenin has benefits on flap tissue survival and is useful in plastic surgery [12]. Previous in vitro studies have demonstrated the usefulness of naringenin and apigenin in protecting the skin from the invasion of UV, tumors, and aging, indicating the potential protective effects of naringenin and apigenin against skin damage. Given the complex metabolic processes in body, in vitro evidence cannot be directly equated to obtaining an in vivo conclusion. Thus, more studies are required to confirm whether these two compounds could exhibit similar or even superior efficacy when administered as dietary supplements.

An increasing number of reports have demonstrated the close relationship between dietary habits and skin metabolism [13,14]. The adverse effect of milk and foods with a high glycemic and/or fat index on skin condition has been verified, showing that these foods promote the development of acne vulgaris [15]. The effect of lifestyle factors on carotenoid levels in the skin demonstrates that a positive relationship exists between increased antioxidant and inflammatory levels and intake of carotenoid-rich foods [16]. Dietary supplementation of plant-derived composition can defend against skin aging after exposure to UV radiation [6,17].

Oleic acid (OA) is regarded as the main trigger for inflammation and oxidation, and sensitivity to this free fatty acid plays a vital role in the pathogenesis of skin disease; the effect of OA has been demonstrated in a mouse model of skin damage caused by over-secretion of skin lipid [18]. The activation of the transcription factor nuclear factor (erythroid-derived 2)-like 2 (Nrf2) is an important mechanism for controlling stress responses and antioxidant defense in organs, including the skin. Nuclear factor-kappa B (NF-κB) is the most important signaling factor for regulating inflammatory responses in mammals and causes the transcription of pro-inflammatory mediators, such as interleukin (IL)-6, tumor necrosis factor-α (TNF-α), IL-1, and intracellular adhesion cyclooxygenase-2 (COX2). Previous studies have shown that naringenin and apigenin regulate the Nrf2 and NF-—κB pathway to defend against oxidative and inflammatory processes [19,20].

Thus, the objective of this study is to explore the protective effects of naringenin and apigenin in improving skin damage and to compare their underlying mechanisms of action. We analyzed the contents of the main lipid components to evaluate sebum production in sebaceous glands. The skin levels of inflammatory factors and antioxidative stress were determined. Histopathological examination was used to evaluate skin injury and inflammatory responses in the back of mice. mRNA expression related to inflammation and antioxidation was further determined to compare the mechanisms of naringenin and apigenin supplementation.

## 2. Materials and Methods

### 2.1. Reagents and Materials

Naringenin (NAR, purity ≥ 97%) derived from *C.* L.Osbeck was obtained from Chengdu Herbpurify Co., Ltd. (Chengdu, China). Apigenin (API, purity ≥ 98%) was derived from *O. javanica* (Blume) DC and purchased from Aladdin^®^ Co., Ltd. (Shanghai, China). Cryptotanshinone (CRY, extracted from Salvia miltiorrhiza, purity ≥ 98%) was obtained from Chengdu Herbpurity Co., Ltd. (Chengdu, China). Naringin and apigenin were dissolved in methyl alcohol and determined using UHPLC-MS/MS (Q Exactive, Thermo Fisher Scientific, Waltham, MA, USA) with a T3 column (2.1 × 100 mm, 1.8 µm) at an operating temperature of 30 °C according to the methods previously reported [21] (Figure S1). Liquid paraffin and oleic acid (OA) were purchased from Sinopharm Chemical Reagent Co., Ltd. (Shanghai, China). Other reagents were purchased from Sinopharm Chemical Reagent Co., Ltd. (Shanghai, China). Enzyme-linked immunosorbent assay (ELISA) kits were provided by Enzyme-linked Biotech Co., Ltd. (Shanghai, China).

Naringenin (NAR, purity ≥ 97%) derived from *C.* L.Osbeck was obtained from Chengdu Herbpurify Co., Ltd. (Chengdu, China). Apigenin (API, purity ≥ 98%) was derived from *O. javanica* (Blume) DC and purchased from Aladdin^®^ Co., Ltd. (Shanghai, China). Cryptotanshinone (CRY, extracted from Salvia miltiorrhiza, purity ≥ 98%) was obtained from Chengdu Herbpurity Co., Ltd. (Chengdu, China). Naringin and apigenin were dissolved in methyl alcohol and determined using UHPLC-MS/MS (Q Exactive, Thermo Fisher Scientific, Waltham, MA, USA) with a T3 column (2.1 × 100 mm, 1.8 µm) at an operating temperature of 30 °C according to the methods previously reported [21] (Figure S1). Liquid paraffin and oleic acid (OA) were purchased from Sinopharm Chemical Reagent Co., Ltd. (Shanghai, China). Other reagents were purchased from Sinopharm Chemical Reagent Co., Ltd. (Shanghai, China). Enzyme-linked immunosorbent assay (ELISA) kits were provided by Enzyme-linked Biotech Co., Ltd. (Shanghai, China).

### 2.2. Animal Experimental Design

C57BL/6 mice (male, 6 weeks old, 20 ± 2 g) were purchased from Charles River Laboratory Animal Technology Co., Ltd. (Jiaxing, China) and fed with commercial chow and sterile water. After 7 days of adaptive feeding, all mice were randomly grouped into 7 groups (n = 6 for each group). Naringenin and apigenin were dissolved in normal saline with Tween-80 (0.5%, *w/v*). The treatment of mice in each group was shown in Table S1. The mice in the control and model groups were orally administrated normal saline with Tween-80 (0.5%, *w/v*). After administration via gavage for a week, 0.1 mL of oleic acid (80% *w/v*) was smeared on the mice’s back once a day to build a skin injury model, which lasted for two weeks. The primary lipophilic extract derived from *Salvia miltiorrhiza*, known as CRY, is a main ingredient in ameliorating skin damage and regulating skin lipids balance and was used as the positive control (10 mg/kg/d). The animal experiments were performed with the approval of the Animal Experimentation Ethics Committee of Jiangnan University (JN.No20220717c0959628[021]).

### 2.3. Observation Index and Skin Damage Scores

Skin damage scores were calculated based on a previous study [22]. The main symptoms included erythema, scales, erosion, and pruritus. Scores of (0–3) were given to evaluate skin damage. A score of 0 was given when there were no symptoms. A score of 1 (mild) was given where there were symptoms including minor erythema, erosion, and pruritus, as well as sparse scales. A score of 2 (moderate) was given to symptoms including major erythema, erosion, and pruritus, as well as a wide range of scales and even scabs. A score of 3 (severe) was given to symptoms including full-scale erythema, scales (scabs), erosion, and pruritus.

### 2.4. Histological Analysis of Skin Tissue

Skin tissues were stained using hematoxylin and eosin (H&E) for histological analysis. The staining process included collection, dehydration, embedment, slicing, and staining. The tissues were observed according to a previous report with minor revisions [23].

### 2.5. Determination of Skin Lipids

The levels of skin total triglycerides (TG), non-esterified fatty acids (NEFA), and cholesterol (CHO) in skin tissues were determined according to the instructions of the kits used (Nanjing Jiancheng Bioengineering Institute, Nanjing, China). Absorption values were obtained using a microplate readers with the skanit software 4.1 (Thermo, Waltham, MA, USA).

### 2.6. Assessment of Antioxidant Activities in Skin Tissue

Antioxidant activities in skin lesions were characterized via the determination of the levels of malondialdehyde (MDA), superoxide dismutase (SOD), catalase (CAT), lipid peroxides (LPO), total antioxidant capacity (T-AOC) and aspartate aminotransferase (AST). The determination was performed according to the instructions of the kits used (Nanjing Jiancheng Bioengineering Institute, Nanjing, China). The obtained absorption values were processed using a one-way analysis of variance (ANOVA) via the SPSS software ver. 22.0 (IBM, Armonk, NY, USA).

### 2.7. Cytokine Measurements Using ELISA Assay

The IL-1β, IL-6, TNF-α, and IL-10 levels of the supernatants from skin lesions were analyzed using ELISA kits (R&D Systems, Minneapolis, MN, USA) according to the manufacturer’s instructions.

### 2.8. Quantitative RT-PCR Analysis

Total RNA was extracted from cells and then reversely transcribed to cDNA using commercial kits (Vazyme, Nanjing, China). RT-PCR amplifications were carried out in a StepOnePlus RT-PCR system (Thermo, Waltham, MA, USA) using the qPCR kit (Vazyme, Nanjing, China). The predesigned gene-specific primers are listed in Table S2.

### 2.9. Immunohistochemical Analysis

Paraffin sections were heated in an oven at 90 °C until the wax fused and then successfully immersed in xylene and gradient ethanol. The tissue sections were hydrated and then incubated with 3% hydrogen peroxide for 25 min, rinsed subsequently with phosphate-buffered saline (PBS) three times, and sealed with 10% goat serum for 45 min. The sections were then incubated overnight with antibodies against NF-κB (1:200, Arigo, Shanghai, China) at 4 °C. After hematoxylin staining, they were sealed with neutral resin and observed under a microscope. The obtained pictures were analyzed using the Image J software, and the expression of NF-κB was analyzed statically using GraphPad Prism 7.0 (GraphPad, La Jolla, CA, USA).

### 2.10. Statistical Analysis

Data analysis was performed to indicate significant difference using a one-way analysis of variance (ANOVA) via the SPSS software ver. 22.0 (IBM, Armonk, NY, USA). The Student–Newman–Keuls test was used for multiple comparisons using GraphPad Prism 7.0 (GraphPad, La Jolla, CA, USA). All data were presented as mean ± standard deviation (SD). *p* < 0.05 was considered statistically significant.

## 3. Results

### 3.1. Determination of Naringenin and Apigenin Based on HPLC-MS/MS

HPLC-MS/MS was used to determine the purity of naringenin and apigenin, and the chromatogram is presented in Figure S1. The peak times of naringenin and apigenin were 6.42 and 6.40 min, respectively, with obvious peak heights. The *m/z* values of naringenin and apigenin (273.07 and 271.05, respectively) were obtained after the MS analysis.

### 3.2. Effects of Naringenin and Apigenin on Body Weight and Food and Water Intake of Mice

**Figure 1:**
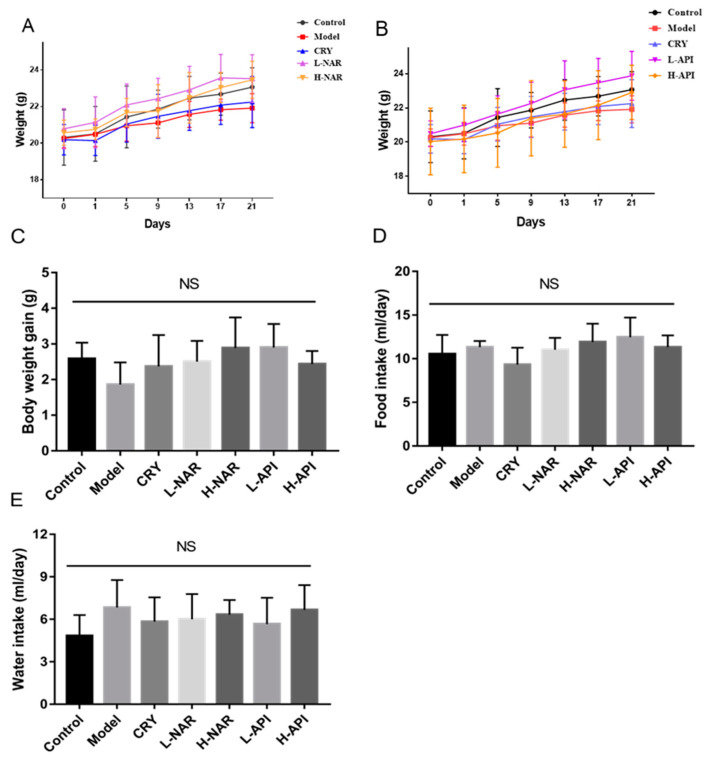
Influences of naringenin and apigenin pretreatments on body weight and food and water intake in mice of different groups. Body weight gain of mice with naringenin pretreatment (**A**) and apigenin pretreatment (**B**); body weight gain of mice in different groups (**C**); and food intake (**D**) and water intake (**E**) of mice in different groups. CRY: cryptotanshinone; L-NAR: naringenin with a dose of 5 mg/kg/d; H-NAR: naringenin with a dose of 10 mg/kg/d; L-API: apigenin with a dose of 5 mg/kg/d; H-API: apigenin with a dose of 10 mg/kg/d; “NS” means no significant differences (*p* > 0.05) among different groups.

The body weight changes in the mice with the pretreatments of naringenin (Figure 1A) and apigenin (Figure 1B) were determined after 21 days of feeding. The body weight gain in the OA-treated mice slightly increased with the intake of naringenin and apigenin compared to that in the model group, whereas the body weight gain in all other groups showed no significant differences (*p* > 0.05; Figure 1C). The food intake and water intake of the mice in all groups were also determined, and the results are shown in (Figure 1D,E) with no significant difference (*p* > 0.05). These results obtained for food and water intake were not related to the interventions with naringenin and apigenin.

### 3.3. Pathological Morphology of Skin Tissues in Mice

**Figure 2:**
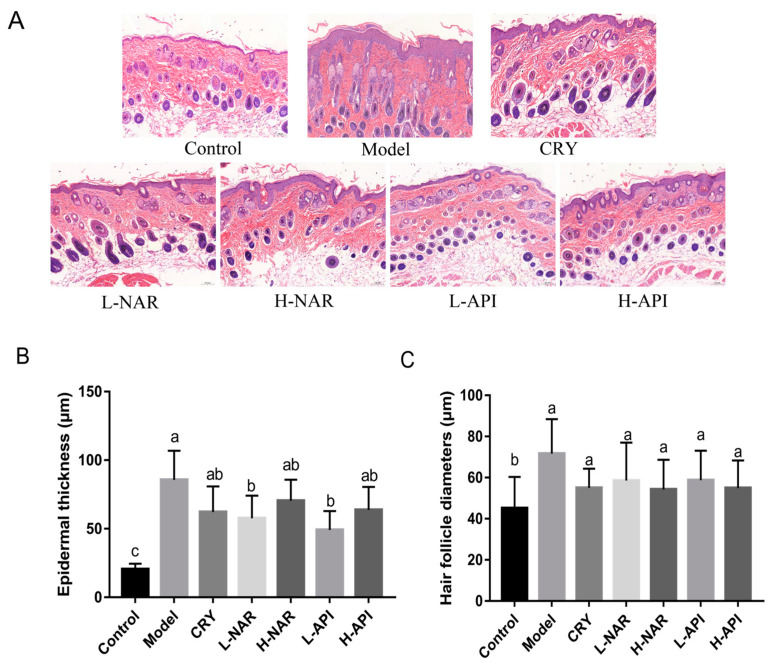
Pathological morphology of skin tissues in mice. Histopathological observation (**A**), epidermal thickness assessment (**B**), and hair follicle diameter assessment (**C**). CRY: cryptotanshinone; L-NAR: naringenin with a dose of 5 mg/kg/d; H-NAR: naringenin with a dose of 10 mg/kg/d; L-API: apigenin with a dose of 5 mg/kg/d; H-API: apigenin with a dose of 10 mg/kg/d. Significant differences (*p* < 0.05) among different groups were presented as “a, b, c”.

The biochemical parameters of the skin tissues from the different groups used to determine injury and inflammation are shown in Figure 2. The hyperkeratosis of skin tissues in the mice treated with OA was characterized by a thickened epidermis, blurred boundaries between epidermis and dermis, collection in hair follicle pores, infiltration of neutrophils in the dermis, and proliferation of sebaceous ducts. The damage scores for the groups are listed in Table 1. However, these pathological conditions were improved in the OA-treated mice with the pretreatments of naringenin and apigenin. The CRY pretreatment improved skin lesions in the OA-treated mice, and a better effect was shown in the naringenin and apigenin groups. The evaluation of epidermal thickness obtained consistent results (Figure 2B and Table 2), and significant differences were detected in the L-NAR and L-API groups compared to the model group (*p* < 0.05). Although there were no differences in hair follicle diameter among the NAR and API groups compared to the model group (*p* > 0.05), the OA treatment resulted in enlarged pores in the model group compared to the control group (*p* > 0.05; Figure 2C and Table 2).

### 3.4. Decreased Skin Sebum Levels with Pretreatment with Naringenin and Apigenin

**Figure 3:**
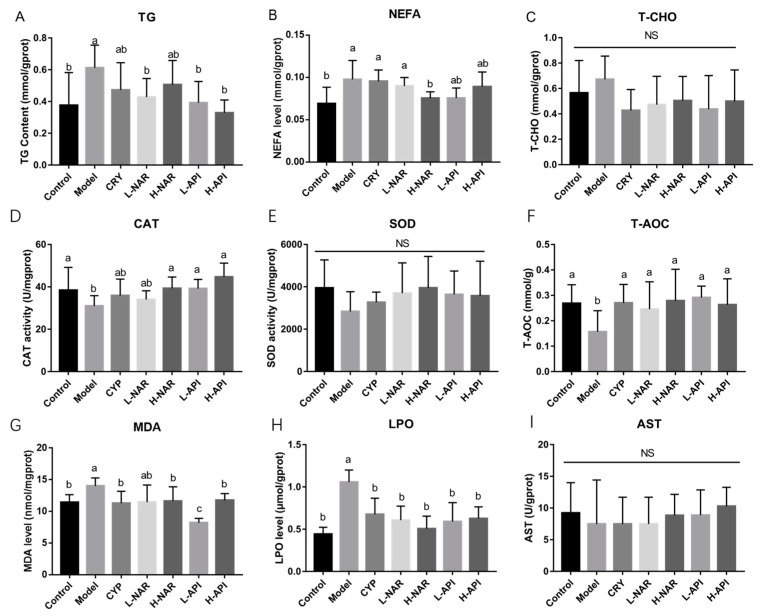
Influences of naringenin and apigenin pretreatments on skin lipids and antioxidative abilities. Skin lipids include total triglyceride (TG; **A**), non-esterified fatty acid (NEFA; **B**), and cholesterol (CHO; **C**) levels in mice skin after the interventions with naringenin and apigenin. Assessment of antioxidative abilities via determining the levels of catalase (CAT; **D**), superoxide dismutase (SOD; **E**), total antioxidant capacity (T-AOC; **F**), malondialdehyde (MDA; **G**), lipid peroxide (LPO; **H**), and aspartate aminotransferase (AST; **I**). CRY: cryptotanshinone; L-NAR: naringenin with a dose of 5 mg/kg/d; H-NAR: naringenin with a dose of 10 mg/kg/d; L-API: apigenin with a dose of 5 mg/kg/d; H-API: apigenin with a dose of 10 mg/kg/d. Significant differences (*p* < 0.05) among different groups were presented as “a, b, c”. Non-significant differences (*p* > 0.05) were presented as “NS”.

The skin TG, NEFA, and T-CHO levels were determined based on pretreatment with naringenin and apigenin, respectively (Figure 3 and Table 2). Apigenin significantly inhibited the TG levels (*p* < 0.05). The TG levels in skin lesions significantly decreased in the L-NAR group (*p* < 0.05), whereas there was no significant difference between the H-NAR and model groups (*p* > 0.05). Although there were no significant differences in the T-CHO levels among the groups (*p* > 0.05), a decreasing trend was observed after the pretreatments with naringenin and apigenin (Figure 3C and Table 2). However, the content of NEFA decreased significantly in the H-NAR group when compared to the model group (*p* < 0.05; Figure 3B and Table 2).

### 3.5. Improved Antioxidative System after Naringenin and Apigenin Supplementation

The increase in the CAT levels is shown in Figure 3D, and apigenin promoted CAT expression when compared to the value in the model group (*p* < 0.05). The SOD levels maintained a similar level in the skin after the naringenin and apigenin pretreatments (*p* > 0.05; Figure 3E and Table 2). The total antioxidative ability was assessed after the T-AOC determination, and a significant increase was observed with the intake of naringenin and apigenin (*p* < 0.05; Figure 3F and Table 2). A remarkable decrease in the MDA level was observed in the H-ANR, L-API, and H-API groups compared to that in the model group (*p* < 0.05), and the MDA level in the L-API group was significantly lower than that in the H-ANR and H-API groups (*p* < 0.05; Figure 3G and Table 2). LPO was determined, and obvious decreases are evident in Figure 3H (*p* < 0.05). Figure 3I exhibits no difference in the AST expression among the groups (*p* > 0.05).

### 3.6. Prevention of Inflammatory Response with Naringenin and Apigenin Pretreatments

**Figure 4:**
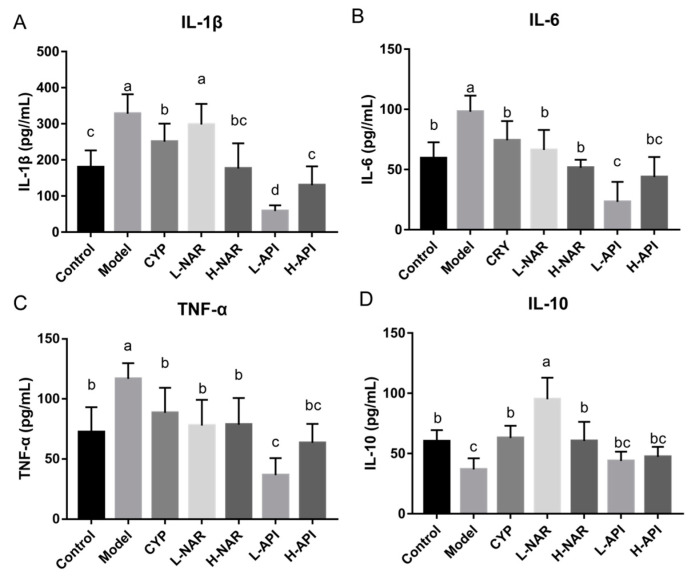
Influences of naringenin and apigenin pretreatments on skin interleukin (IL)-1β, IL-6, tumor necrosis factor (TNF)-α, and IL-10 levels in OA-treated mice. IL-1β expression (**A**), IL-6 expression (**B**), TNF-α expression (**C**), IL-10 expression (**D**). CRY: cryptotanshinone; L-NAR: naringenin with a dose of 5 mg/kg/d; H-NAR: naringenin with a dose of 10 mg/kg/d; L-API: apigenin with a dose of 5 mg/kg/d; H-API: apigenin with a dose of 10 mg/kg/d. Significant differences (*p* < 0.05) among different groups were presented as “a, b, c”.

The levels of IL-6, IL-1β, TNF-α, and IL-10 in the OA-treated mice’s skin were evaluated (Figure 4 and Table 2). The levels of IL-6, IL-1β, and TNF-α significantly decreased with naringenin and apigenin pretreatments (*p* < 0.05). The decreases in the levels of these proinflammatory factors appeared to be dose-dependent with the pretreatment of naringenin; on the contrary, the OA-treated mice in the L-API group were more efficient at alleviating inflammation than those in the H-API group (Figure 4 and Table 2). Naringenin induced an increase in IL-10 expression (*p* < 0.05) compared to that in the model group, and naringenin (10 mg/kg) was thought to be better for IL-10 expression (Figure 4D and Table 2). Apigenin at 5 mg/kg and 10 mg/kg doses significantly reduced IL-10 expression in the skin lesions of the OA-treated mice (Figure 4D and Table 2). These results suggested that naringenin and apigenin alleviated inflammatory response in the OA-treated mice.

### 3.7. Effects of Naringenin and Apigenin on mRNA Levels Related to Antioxidation and Inflammation

**Figure 5:**
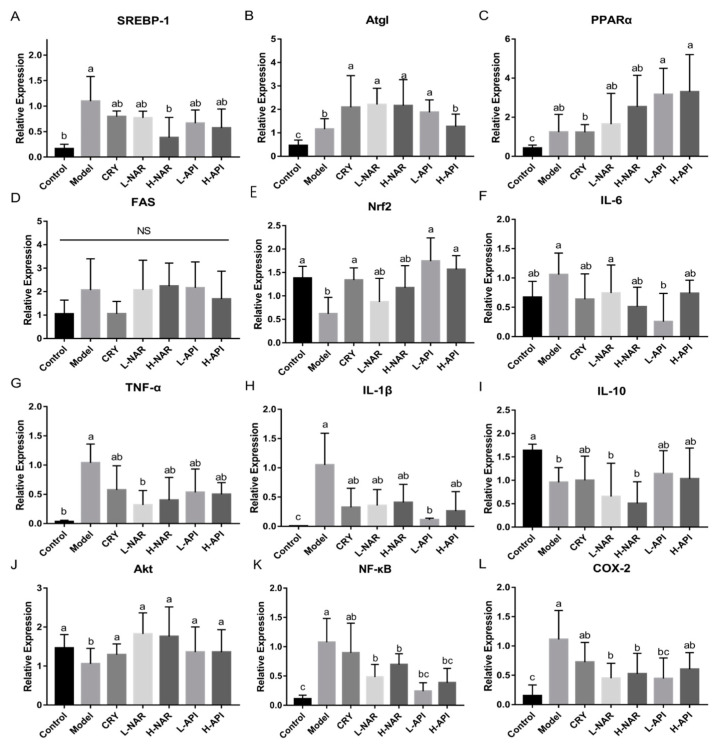
Influences of naringenin and apigenin pretreatments on lipid metabolism and skin inflammation. SREBP-1 expression (**A**), Atgl expression (**B**), PPARα expression (**C**), FAS expression (**D**), Nrf2 expression (**E**), IL-6 expression (**F**), TNF-α expression (**G**), IL-1β expression (**H**), IL-10 expression (**I**), Akt expression (**J**), NF-κB expression (**K**), COX-2 expression (**L**). CRY: cryptotanshinone; L-NAR: naringenin with a dose of 5 mg/kg/d; H-NAR: naringenin with a dose of 10 mg/kg/d; L-API: apigenin with a dose of 5 mg/kg/d; H-API: apigenin with a dose of 10 mg/kg/d; SREBP-1: sterol response element-binding protein-1; Atgl: adipose triacylglyceride lipase; PPARα: peroxisome proliferator-activated receptor α; FAS: fatty acid synthetase; Nrf2: transcription factor nuclear factor (erythroid-derived 2)-like 2; AKT: protein kinase B; NF-κB: nuclear factor-kappa B; COX-2: cyclooxygenase-2. Significant differences (*p* < 0.05) among different groups were presented as “a, b, c”. Non-significant differences (*p* > 0.05) were presented as “NS”.

To further evaluate the influence of naringenin and apigenin pretreatments on the OA-treated mice, the mRNA expression levels of genes related to skin sebum and inflammation were analyzed (Figure 5 and Table 2). The level of sterol response element-binding protein (SREBP) decreased in the naringenin and apigenin pretreatment groups compared to that in the model group (Figure 5A), and similar levels of SREBP were observed in the H-NAR and control groups. Naringenin and apigenin promoted peroxisome proliferator-activated receptor α (PPARα) and adipose triacylglyceride lipase (Atgl) expression, and it seemed that the PPAR levels were dose-dependent (Figure 5B,C and Table 2). However, fatty acid synthetase (FAS) gene expression did not vary significantly among the groups (Figure 5D and Table 2).

Compared to the model group, apigenin increased Nrf2 expression (Figure 5E), but no difference was shown in the NAR group. The mRNA expression of proinflammatory cytokines, including IL-6, TNF-α, and IL-1β, reduced after naringenin and apigenin pretreatments compared to that in the model group (Figure 5F–H and Table 2). A clear reduction in IL-1β and IL-6 was presented in the L-API group, and a significant decrease in TNF-α was shown in the L-NAR group. The naringenin and apigenin pretreatment groups showed no differences in the expression of IL-10 level compared to that in the model group (Figure 5I, Table 2). The mRNA expression of inflammatory factors indicated the importance of relieving inflammation to cure sebum metabolism disorders. Furthermore, protein kinase B (Akt), NF-κB, and COX-2 expressions were determined. Increased Akt level and decreased NF-κB and COX-2 levels were observed after naringenin and apigenin pretreatments in the OA-treated mice (Figure 5J–L and Table 2).

### 3.8. Effects of Naringenin and Apigenin on NF-κB Expression

**Figure 6:**
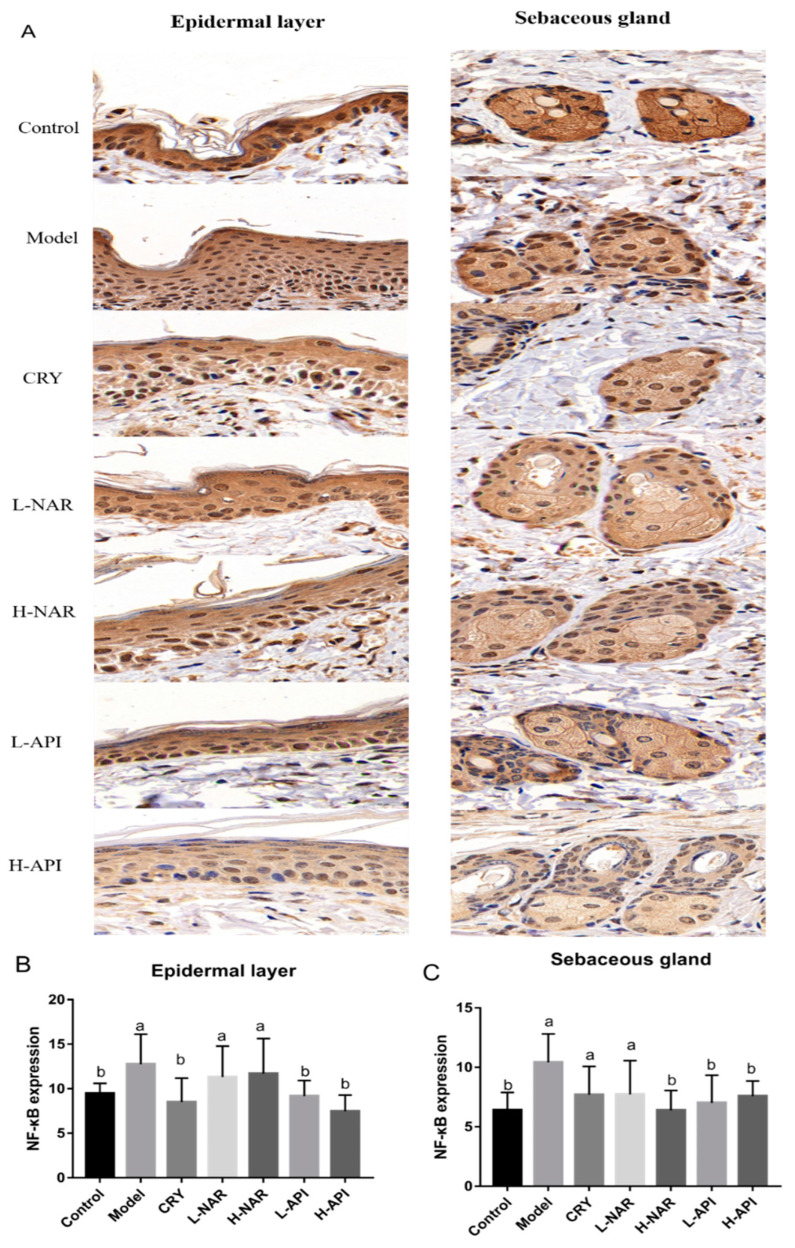
NF-κB expression based on immunohistochemical analysis in the epidermal layer and sebaceous gland. Immunohistochemical photographs of the epidermal layer and sebaceous gland (**A**), and quantitative analysis of NF-κB expression in the epidermal layer (**B**) and in the sebaceous gland (**C**). CRY: cryptotanshinone; L-NAR: naringenin with a dose of 5 mg/kg/d; H-NAR: naringenin with a dose of 10 mg/kg/d; L-API: apigenin with a dose of 5 mg/kg/d; H-API: apigenin with a dose of 10 mg/kg/d. Significant differences (*p* < 0.05) among the different groups were presented as “a, b”. Non-significant differences (*p* > 0.05) were presented as “NS”.

To further confirm whether the expression of NF-κB played an important role in improving skin lesions in the OA-treated mice after being administered naringenin and apigenin, immunohistochemical analysis was performed (Figure 6). NF-κB was expressed in skin issues, mainly in the epidermis and sebaceous glands. A high expression of inflammatory cells in the dermis was also observed in the OA-treated mice. Based on the quantitative analysis, apigenin induced a significantly lower expression of NF-κB in skin issues (*p* < 0.05) than that in the model group (Figure 6 and Figure S3 and Table 2). The epidermal layer appeared to exhibit higher NF-κB expression, which was related to its thickness. The number of sebaceous glands limited the content of NF-κB, although NF-κB levels were presented in all groups.

## 4. Discussion

Ingredients obtained from herbs and edible plants have medicinal and nutritional value and are widely used in medicine, foods, cosmetics, and other fields. Naringenin and apigenin are regarded as two universal molecules that have gained considerable attention; they were used in the present study to explore their effects on the maintenance of skin barriers. The back of mice was smeared with OA after shaving, which is a well-known model for causing multiple layers of tight keratinized cells in the epithelium of hair follicles to block hair follicle pores and promote inflammation [24,25]. Changes in skin lipids affect skin barrier functions, such as palmitic acid, stearic acid, and OA, which penetrate the dermo-epidermal barrier to promote the secretion of inflammatory factors and activate macrophages and related inflammatory and oxidative systems. Thus, it would be beneficial to explore the protective properties of naringenin and apigenin pretreatments against sebum metabolism disorders and related inflammation in OA-treated mice.

The skin barrier was broken and symptoms of erythema, scales, erosion, and pruritus gradually emerged after the OA application in mice. The overall assessment of skin damage showed conspicuous improvements in the skin condition with the naringenin and apigenin pretreatments (*p* < 0.05; Table 1 and Figure S2). Pathological tissue analysis indicated the apigenin pretreatment decreased epidermal thickness and reduced inflammatory invasion. Previous studies have presented that apigenin can repair skin and relieve inflammation [11,12]. In addition, fewer lipid droplets in skin tissues were observed in the API group than in the NAR group, suggesting apigenin may affect sebum balance via regulating skin fat metabolism.

To further determine whether apigenin was more effective than naringin in improving the skin barrier and alleviating inflammation, the main lipid components related to antioxidative and inflammatory factors in the skin were determined. The lipid-lowering ability of apigenin has been reported in vivo and in vitro studies [26,27,28], which is also confirmed in our results showing that apigenin has better effects on reducing TG and NEFA contents. Changes in sebum levels in terms of quality and quantity can induce keratinization abnormality in ductal epithelial cells of hair follicle sebaceous glands, followed inflammation [29]. Fatty acids are highly expressed in the skin lesions of patients with sebum disorders, resulting in increased secretion of inflammatory factors, especially IL-1β, IL-6, and TNF-α [9,24]. The results of this study showed that naringenin and apigenin decreased the NEFA levels and reduced the levels of IL-1β, IL-6, and TNF-α. The evidence suggested that pretreatment with naringenin and apigenin might improve skin lipids issues by inhibiting proinflammatory cytokines. The disruption of sebum balance affects the activities of lipases, such as FAS, hormone-sensitive lipase (Hsl), and Atgl [30,31,32,33]. FAS, Atgl, and Hsl are responsible for fatty acid synthesis, TG hydrolysis, and lipolysis [25,33]. FAS is also related to immune activation, and blocked FAS expression can attenuate obesity-induced adipose tissue inflammation by inhibiting pro-inflammatory pathways [34]. Atgl is a major enzyme involved in adipose tissue TG catabolism, and ATGL is downregulated in mouse models of obesity [30]. There were no significant changes in the FAS levels; however, the mRNA level of Atgl significantly increased, explaining the lower levels of TG contents after the naringenin and apigenin pretreatments in the OA-treated mice. To understand the increases in Atgl levels, especially in the NAR group, related key proteins were analyzed. Sebum improvement has been reported to be associated with SREBP-1 and PPAR expressions [35,36]. SREBP-1 is a lipogenesis-related factor that maintains the balance in glucose metabolism and cholesterol and lipid metabolism in vivo [33]. PPARs participate in various processes, including improving endothelial function, promoting CHO transfer, regulating glucose and lipid metabolism, and inhibiting inflammation [37,38,39]. Some reports have illustrated that decreased SREBP-1 and PPARα expressions regulate a low level of lipids in human sebocytes [33]. For the naringenin pretreatment, the lower mRNA expression of SERBP-1 than that in the model group indicated naringenin might regulate sebum depending on fat metabolism via the SREBP-1/ATGL pathway.

In addition, a trial demonstrated that the skin surface of patients with acne presented a mass of lipoperoxides owing to squalene peroxidation and a reduction in antioxidant sebum (vitamin E) [40]. This indicates that oxidized lipids play an important role in the development of skin lesions. Naringenin reduces UVB-induced oxidative stress in the skin of hairless mice [6]. A study demonstrated that T-AOC and NRF were improved in apigenin-treated aged mice, thereby inhibiting oxidative stress [41]. Apigenin restores the viability of UV-treated normal human dermal fibroblasts by altering their anti-inflammatory and antioxidant properties [26]. Naringenin and apigenin improved the antioxidative system in the skin, decreased MDA and LPO levels, and increased CAT and T-AOC levels. Significantly increased expression of Nrf2 level was observed after the naringenin and apigenin pretreatments. The Nrf2 pathway mediates the oxidative system and ultimately exerts anti-inflammatory effects. The transcription factor Nrf2 and its principal negative regulator, the E3 ligase adaptor Kelch-like ECH-associated protein 1, play a pivotal role in maintaining intracellular redox homeostasis and regulating inflammation [42]. In addition, the crosstalk between the Nrf2 pathway and NF-κB expression has been demonstrated. NF-κB is a key biomarker involved in various inflammatory diseases, such as arthritis, carditis, folliculitis, enteritis, and pneumonia. We focused on the exploration of the NF-κB pathway, and NF-κB pathway is a common, central pathway involved in promoting the formation of pro-inflammatory and pro-labor mediators in tissues [43,44]. NF-κB protein is located in the cytoplasm and can be activated by several different stimuli. The activation of NF-κB is expressed via the positive and negative regulations of many important genes involved in the essential inflammatory process, including chemokines and proinflammatory cytokines. PPARs block this activation and reduce the synthesis and secretion of inflammatory molecules. IL-1β, IL-6, and TNF-α are triggers of NF-κB [43]. Skin inflammation is suppressed via the baicalin-regulated NF-κB/MAPK signaling pathway [45]. The migration of neutrophils and lymphocytes and the activation of NF-κB were found to be present in patients with seborrheic dermatitis [46]. The Akt pathway also plays an important role in the development of inflammation, and Akt activation downregulates NF-κB expression, thus affecting the NF-κB pathway [47]. Previous studies demonstrated that inflammatory diseases were improved by mediating Akt/NF-κB pathway signaling [43]. Consistently, downregulation of NF-κB and upregulation of Akt were observed after the naringenin and apigenin pretreatments compared to the levels in the model group. Furthermore, the decreased mRNA levels of IL-1β, IL-6, and TNF-α were consistent. COX-2 expression was also downregulated after the naringenin and apigenin pretreatments in the OA-treated mice. COX-2 is thought to be an inflammatory inducer, with high expression of COX-2 found in inflammatory regions [48]. In general, COX-2 is not expressed in most tissues but is highly expressed in damaged and inflamed tissues [49,50]. Previous studies showed that COX-2 expression was regulated by not only proinflammatory cytokines but also NF-κB levels [50]. Notably, NF-κB has two binding sites in the promoter of COX-2, and increases in COX-2 are induced after specific changes in these binding sites, indicating that the transcriptional activation of COX-2 requires the participation of NF-κB [50]. When drugs were applied in acute lung injury, the conditions were improved following decreased expression of COX-2 and NF-κB levels [51]. The decreases in COX-2 level reflected the reduction in NF-κB expression, suggesting the potential therapeutic effects of naringenin and apigenin in regulating NF-κB signaling.

The present study demonstrated that naringenin and apigenin can ameliorate skin damage by regulating the Nrf2 and NF-κB pathways to improve antioxidative stress and anti-inflammatory response in OA-induced mice, indicating naringenin and apigenin as potential dietary supplements to protect against skin damage. In addition, fruits and vegetables rich in apigenin and naringin could be consumed in a more appropriate way. The information provided in this study is useful to the development of new health-promoting diets and can play an important role in the improvement of human health. However, more studies on phytochemicals and their healthy components are required to further explore their benefits for health.

## 5. Conclusions

Pretreatments with naringenin and apigenin alleviated skin damage in the OA-induced mice by decreasing skin lipid levels, improving keratinization and antioxidation, and reducing the release of proinflammatory factors. The improvement in antioxidative abilities was also mediated by the activation of the Nrf2 pathway, and the reduction in inflammation was regulated by naringenin and apigenin via NF-κB signaling.

## Figures and Tables

**Table 1:** Assessment of skin damage among groups.

Symptoms	Control	Model	CRY	L-NAR	H-NAR	L-API	H-API
Erythema	0	1.38 ± 0.52 ^a^	0.43 ± 0.53 ^a^	0.25 ± 0.46 ^a^	0.75 ± 0.46 ^a^	0.25 ± 0.46 ^a^	0.25 ± 0.46 ^a^
Scales	0	2.25 ± 0.71 ^a^	2.14 ± 0.69 ^a^	1.13 ± 0.64 ^a^	1.50 ± 0.83 ^a^	1.00 ± 0.53 ^a^	1.50 ± 0.53 ^a^
Erosion	0	0.50 ± 0.53 ^a^	0.29 ± 0.49 ^a^	0.13 ± 0.35 ^a^	0.38 ± 0.74 ^a^	0.13 ± 0.35 ^a^	0.25 ± 0.46 ^a^
Pruritus	0	1.50 ± 0.53 ^a^	1.29 ± 0.49 ^a^	1.13 ± 0.35 ^a^	1.25 ± 0.46 ^a^	1.13 ± 0.35 ^a^	1.13 ± 0.35 ^a^
Total	0	5.63 ± 1.41 ^a^	4.14 ± 1.77 ^b^	2.63 ± 0.74 ^bc^	3.88 ± 1.13 ^bc^	2.38 ± 0.92 ^c^	3.25 ± 1.04 ^bc^

All data are presented as mean ± standard deviation (SD). “a, b, c”: different letters indicate significant differences (*p* < 0.05) among different groups.

**Table 2:** The results and significance of the main parameters among the control, model, CRY, L-NAR, H-NAR, L-API, and H-API groups.

	Group	Control	Model	CRY	L-NAR	H-NAR	L-API	H-NAR
Parameter								
Apparent symptoms	Damage scores	0	5.63 ± 1.41 ^a^	4.14 ± 1.77 ^b^	2.63 ± 0.74 ^bc^	3.88 ± 1.13 ^bc^	2.38 ± 0.92 ^c^	3.25 ± 1.04 ^bc^
	Epidermal thickness (μm)	20.5 ± 1.44 ^c^	85.68 ± 6.55 ^a^	62.37 ± 7.50 ^ab^	57.67 ± 5.45 ^b^	70.45 ± 5.84 ^ab^	63.85 ± 5.91 ^b^	49.24 ± 4.83 ^ab^
	Hair follicle diameter (μm)	45.09 ± 6.23 ^b^	71.73 ± 6.84 ^a^	55.07 ± 3.79 ^a^	58.63 ± 7.50 ^a^	50.97 ± 5.89 ^a^	51.63 ± 5.86 ^a^	58.79 ± 5.45 ^a^
Skin lipid expression	TG level	0.38 ± 0.08 ^b^	0.61 ± 0.06 ^a^	0.47 ± 0.07 ^ab^	0.43 ± 0.05 ^b^	0.51 ± 0.06 ^ab^	0.39 ± 0.05 ^b^	0.33 ± 0.03 ^b^
	NEFA level	0.069 ± 0.008 ^b^	0.098 ± 0.009 ^a^	0.095 ± 0.005 ^a^	0.090 ± 0.004 ^a^	0.075 ± 0.003 ^b^	0.076 ± 0.007 ^ab^	0.089 ± 0.007 ^ab^
	T-CHO level	0.57 ± 0.10 ^a^	0.67 ± 0.07 ^a^	0.43 ± 0.07 ^a^	0.47 ± 0.09 ^a^	0.50 ± 0.08 ^a^	0.44 ± 0.11 ^a^	0.50 ± 0.10 ^a^
Antioxidative expression	CAT level	38.43 ± 4.08 ^a^	31 ± 1.84 ^b^	35.91 ± 2.94 ^ab^	34.09 ± 1.55 ^ab^	39.34 ± 2.01 ^a^	39.21 ± 1.62 ^a^	44.7 ± 2.47 ^a^
	SOD level	3948 ± 541.2 ^a^	2839 ± 380.4 ^a^	3273 ± 195 ^a^	3701 ± 585.1 ^a^	3954 ± 605.3 ^a^	3641 ± 452.3 ^a^	3577 ± 666.9 ^a^
	T-AOC level	0.27 ± 0.03 ^a^	0.16 ± 0.03 ^b^	0.27 ± 0.03 ^a^	0.25 ± 0.04 ^a^	0.28 ± 0.04 ^a^	0.29 ± 0.02 ^a^	0.26 ± 0.04 ^a^
	MDA level	11.48 ± 0.49 ^b^	14.02 ± 0.52 ^a^	11.31 ± 0.77 ^b^	11.49 ± 1.10 ^ab^	11.68 ± 0.90 ^b^	8.23 ± 0.28 ^c^	11.75 ± 0.44 ^b^
	LPO level	0.44 ± 0.03 ^b^	1.06 ± 0.06 ^a^	0.68 ± 0.08 ^b^	0.61 ± 0.07 ^b^	0.51 ± 0.06 ^b^	0.59 ± 0.09 ^b^	0.63 ± 0.06 ^b^
	AST level	9.22 ± 1.81 ^a^	7.49 ± 2.83 ^a^	7.49 ± 1.49 ^a^	7.49 ± 1.49 ^a^	8.85 ± 1.18 ^a^	8.87 ± 1.41 ^a^	10.28 ± 1.06 ^a^
Inflammatory cytokines	Skin IL-6 (pg/mg protein)	59.45 ± 5.43 ^c^	98.01 ± 5.50 ^a^	74.26 ± 6.55 ^b^	66.37 ± 6.74 ^a^	51.57 ± 2.68 ^bc^	23.24 ± 6.76 ^d^	43.87 ± 6.77 ^c^
	Skin IL-1β (pg/mg protein)	179.8 ± 19.01 ^c^	328.2 ± 21.85 ^a^	250.7 ± 20.42 ^b^	298.2 ± 23.24 ^a^	176.4 ± 28.44 ^bc^	58.7 ± 6.36 ^d^	130.1 ± 21.26 ^c^
	Skin TNF-α (pg/mg protein)	72.49 ± 8.45 ^b^	116.8 ± 5.32 ^a^	88.58 ± 8.44 ^b^	77.96 ± 8.70 ^b^	78.63 ± 9.01 ^b^	36.7 ± 5.76 ^c^	63.57 ± 6.40 ^bc^
	Skin IL-10 (pg/mg protein)	60.22 ± 3.79 ^b^	36.76 ± 3.80 ^c^	62.90 ± 4.16 ^b^	95.04 ± 7.32 ^a^	60.43 ± 6.47 ^b^	43.83 ± 3.14 ^bc^	47.25 ± 3.41 ^bc^
Oxidative and lipid metabolism	SREBP-1 mRNA expression	0.16 ± 0.05 ^b^	1.10 ± 0.24 ^a^	0.79 ± 0.06 ^ab^	0.77 ± 0.07 ^ab^	0.38 ± 0.20 ^b^	0.66 ± 0.15 ^ab^	0.57 ± 0.21 ^ab^
	Atgl mRNA expression	0.46 ± 0.12 ^c^	1.16 ± 0.20 ^b^	2.10 ± 0.60 ^a^	2.21 ± 0.31 ^a^	2.16 ± 0.50 ^a^	1.88 ± 0.24 ^a^	1.27 ± 0.24 ^b^
	PPARα mRNA expression	0.42 ± 0.07 ^c^	1.24 ± 0.45 ^ab^	1.23 ± 0.18 ^b^	1.64 ± 0.65 ^ab^	2.53 ± 0.66 ^ab^	3.17 ± 0.55 ^a^	3.30 ± 0.72 ^a^
	FAS mRNA expression	1.05 ± 0.27 ^a^	2.07 ± 0.60 ^a^	1.05 ± 0.24 ^a^	2.07 ± 0.52 ^a^	2.24 ± 0.40 ^a^	2.16 ± 0.45 ^a^	1.69 ± 0.45 ^a^
	Nrf2 mRNA expression	1.38 ± 0.13 ^a^	0.62 ± 0.18 ^b^	1.34 ± 0.13 ^a^	0.87 ± 0.25 ^ab^	1.18 ± 0.24 ^ab^	1.75 ± 0.25 ^a^	1.57 ± 0.15 ^a^
Inflammation signaling pathway	Relative Akt mRNA expression	1.46 ± 0.47 ^a^	1.06 ± 0.20 ^b^	1.30 ± 0.14 ^a^	1.83 ± 0.77 ^a^	1.76 ± 0.38 ^a^	1.36 ± 0.29 ^a^	1.36 ± 0.23 ^a^
	Relative COX-2 mRNA expression	0.15 ± 0.08 ^c^	1.11 ± 0.02 ^a^	0.73 ± 0.14 ^ab^	0.45 ± 0.10 ^b^	0.53 ± 0.14 ^b^	0.45 ± 0.14 ^bc^	0.60 ± 0.11 ^ab^
	Relative NF-κB mRNA expression	0.11 ± 0.03 ^c^	1.08 ± 0.20 ^a^	0.90 ± 0.25 ^ab^	0.48 ± 0.11 ^b^	0.70 ± 0.09 ^b^	0.24 ± 0.07 ^bc^	0.39 ± 0.12 ^bc^
	Relative IL-1β mRNA expression	0.01 ± 0.003 ^c^	1.05 ± 0.27 ^a^	0.33 ± 0.16 ^ab^	0.36 ± 0.14 ^ab^	0.41 ± 0.15 ^ab^	0.11 ± 0.01 ^b^	0.26 ± 0.17 ^ab^
	Relative IL-6 mRNA expression	0.67 ± 0.14 ^ab^	1.06 ± 0.18 ^a^	0.64 ± 0.22 ^ab^	0.74 ± 0.24 ^a^	0.51 ± 0.17 ^ab^	0.26 ± 0.24 ^b^	0.74 ± 0.11 ^ab^
	Relative IL-10 mRNA expression	1.64 ± 0.07 ^a^	0.96 ± 0.16 ^b^	1.00 ± 0.26 ^ab^	0.65 ± 0.36 ^b^	0.51 ± 0.23 ^b^	1.42 ± 0.45 ^ab^	1.03 ± 0.33 ^ab^
	Relative TNFα mRNA expression	0.04 ± 0.01 ^b^	1.04 ± 0.16 ^a^	0.58 ± 0.21 ^ab^	0.32 ± 0.12 ^b^	0.40 ± 0.19 ^ab^	0.53 ± 0.20 ^ab^	0.50 ± 0.10 ^ab^
Immunohistochemical analysis	NF-κB expression in skin issues	9.806 ± 0.76 ^b^	16.56 ± 0.62 ^a^	14.3 ± 0.65 ^b^	12.48 ± 0.84 ^b^	13.02 ± 0.84 ^b^	11.18 ± 0.88 ^b^	11.32 ± 1.07 ^b^
	NF-κB expression in the epidermal layer	9.47 ± 0.46 ^b^	12.76 ± 1.37 ^a^	8.51 ± 1.10 ^b^	11.32 ± 1.42 ^a^	11.7 ± 1.61 ^a^	9.18 ± 0.71 ^b^	7.48 ± 0.74 ^b^
	NF-κB expression in the sebaceous gland	6.41 ± 0.60 ^b^	10.41 ± 0.98 ^a^	7.71 ± 1.38 ^a^	7.23 ± 1.16 ^a^	6.39 ± 0.68 ^b^	7.02 ± 0.95 ^b^	7.59 ± 0.52 ^b^

All data are presented as mean ± standard deviation (SD). “a, b, c”: different letters indicate significant differences (*p* < 0.05) among different groups.

## Data Availability

Data are contained within the article.

## Supplementary Materials

The following supporting information can be downloaded at https://www.mdpi.com/article/10.3390/foods12112120/s1. Figure S1: Chromatogram of naringenin and apigenin based on LC-MS/MS: chromatogram of apigenin (A) and chromatogram of naringenin (B). Figure S2: Overall assessment score of skin injury in mice with OA treatment. Scores were given by evaluating symptoms such as skin erythema, scales, erosion, and pruritus. Single symptoms were divided into 4 levels: a 0 score for asymptomatic effects, 1 for mild symptoms, 2 for moderate symptoms, and 4 for severe symptoms. Figure S3: (A,B) NF-κB expression based on immunohistochemical analysis in skin issues. Significant differences (*p* < 0.05) among different groups were presented as “a, b, c”. Non-significant differences (*p* > 0.05) were presented as “NS”. Table S1: List of treatments in different groups. Table S2: Primer sequences for the quantitative real-time PCR (qRT-PCR) analysis used in the present study.
