# Supplementary material for: Protective Effects of Naringenin and Apigenin in Ameliorating Skin Damage via Mediating the Nrf2 and NF-κB Pathways in Mice
Source: Foods. 2023 May 24;12(11):2120. doi: 10.3390/foods12112120 (PMC10252862; doi:10.3390/foods12112120)
Supplement: Supplementary file 1 [file foods-12-02120-s001.zip › foods-2388583-SI.pdf]

Supplementary Material

Protective Effects of Naringenin and Apigenin on Ameliorating Skin Damage via Mediating Nrf2 and NF-κB pathway in Mice

Jie Li[1,2], Bingyong Mao[1,2], Xin Tang[1,2], Qiuxiang Zhang[1,2], Jianxin Zhao[1,2], Hao Zhang[1,2,3,*], Shumao Cui[1,2]

[1] State Key Laboratory of Food Science and Technology, Jiangnan University, Wuxi 214122, China 1; 7210112021@stu.jiangnan.edu.cn (J.L.); maobingyong@jiangnan.edu.cn (B.M.); xintang@jiangnan.edu.cn (X.T.); zhangqx@jiangnan.edu.cn (Q.Z.); zhaojianxin@jiangnan.edu.cn (J.Z.); zhanghao61@jiangnan.edu.cn (H.Z.); cuishumao@jiangnan.edu.cn (S.C.);
[2] School of Food Science and Technology, Jiangnan University, Wuxi 214122, China
[3] National Engineering Research Center for Functional Food, Jiangnan University, Wuxi 214122, China
* Correspondence: zhanghao61@jiangnan.edu.cn

This file includes:

Figure S1. Chromatogram of naringenin and apigenin by LC-MS/MS. Chromatogram of apigenin (A); Chromatogram of naringenin (B).

Figure S2. Overall assessment score of skin injury in mice with OA treatment. The scores were given by evaluating symptoms such as skin erythema, scales, erosion and pruritus. Single symptom was divided into four levels, the 0 score for asymptomatic effects,1 for the mild symptom, 2 for the moderate symptom and 4 was responsible for the severe injury.

Figure S3. NF-κB expression with immunohistochemical analysis in skin issues. The significant differences ($P < 0.05$) among different groups were presented as "a, b, c"; The no significant differences ($P > 0.05$) were presented as "NS".

Table S1. List of treatments in different groups.

Table S2. Primer Sequences for Quantitative real time PCR (qRT-PCR) analysis used in the present study.

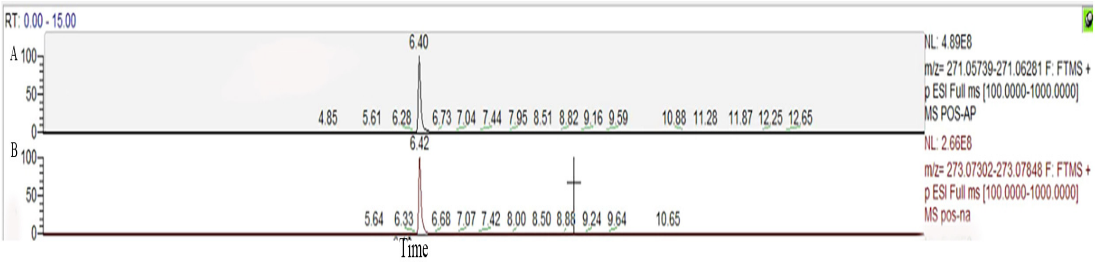

Figure S1. Chromatogram of naringenin and apigenin by LC-MS/MS. Chromatogram of apigenin (A); Chromatogram of naringenin (B).

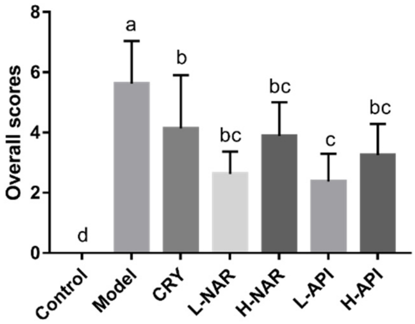

Figure S2. Overall assessment score of skin injury in mice with OA treatment. The scores were given by evaluating symptoms such as skin erythema, scales, erosion and pruritus. Single symptom was divided into four levels, the 0 score for asymptomatic effects,1 for the mild symptom, 2 for the moderate symptom and 4 was responsible for the severe injury.

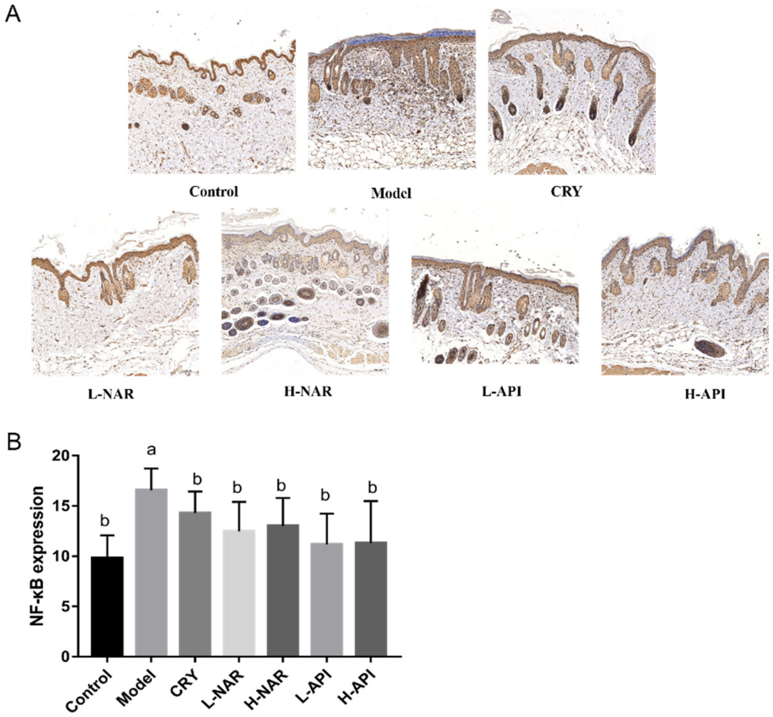

Figure S3. NF-κB expression with immunohistochemical analysis in skin issues. The significant differences (P < 0.05) among different groups were presented as "a, b, c"; The no significant differences (P >0.05) were presented as "NS".

Table S1. List of treatments in different groups.

Treatment \ Groups		Control (n=6)	Model (n=6)	CRY (n=6)	L-NAR (n=6)	H-NAR (n=6)	L-API (n=6)	H-API (n=6)
OA Treatment		-	+	+	+	+	+	+
Cryptotanshinone Intervention	10mg/kg/d	-	-	+	-	-	-	-
Naringin Intervention	5 mg/kg/d	-	-	-	+	-	-	-
	10mg/kg/d	-	-	-	-	+	-	-
Apigenin Intervention	5 mg/kg/d	-	-	-	-	-	+	-
	10mg/kg/d	-	-	-	-	-	-	+

"+" means the treated mice in group. "-" means the non-treated mice in group.

Table S2. Primer Sequences for Quantitative real time PCR (qRT-PCR) analysis used in the present study.

Gene	Forward (5′ to 3′)	Reverse (5′ to 3′)
GAPDH	GTATGACTCCACTCACGGCAAA	GGTCTCGCTCCTGGAAGATG
SREBP-1	GATGTGCGAACTGGACACAG	CATAGGGGGCGTCAAACAG
Atgl	CTTCCTCGGGGTCTACCACA	GCCTCCTTGGACACCTCAATAA
PPARα	TATTCGGCTGAAGCTGGTGTAC	CTGGCATTTGTTCCGGTTCT
Fas	GGCCCCTCTGTTAATTGGCT	GGATCTCAGGGTTGGGGTTG
Nrf2	CTCAGCATGATGGACTTGGA	TCTATGTCTTGCCTCCAAAGG
IL-1β	TGGGCCTCAAAGGAAAGAAT	CAGGCTTGTGCTCTGCTTGT
IL-6	ACAACCACGGCCTTCCCTACTT	CACGATTTCCCAGAGAACATGTG
TNF-α	ACCCTCACACTCAGATCATCTTC	TGGTGGTTTGCTACGACGT
IL-10	TCGTTTGTACCTCTCTCCGA	CTTCCCAAGGAAGAACCCC
Akt	CTCATTCCAGACCCACGACC	TAGGAGAACTTGATCAGGCGG
NF-κB	ATGGCAGACGATGATCCCTA	TGATGGGCCTTCACACACATA-
COX-2	CCCAGAGCTCCTTTTCAACC	ATTTGGCACATTTCTTCCCC
